# Exercise and Ferroptosis in Neurodegenerative Diseases: Direct Evidence, Mechanistic Links, and Translational Gaps

**DOI:** 10.3390/ijms27156628

**Published:** 2026-07-25

**Authors:** Mengzhao Han, Haoran Huang, Xinguo Yuan, Penglei Fan, Ming Li

**Affiliations:** College of Education and Sports Sciences, Yangtze University, Jingzhou 434023, China; hanmengzhao.stu@yangtzeu.edu.cn (M.H.); yuanxinguo@yangtzeu.edu.cn (X.Y.)

**Keywords:** ferroptosis, exercise, neurodegenerative disease, iron homeostasis, lipid peroxidation, muscle–brain axis

## Abstract

Ferroptosis is an iron-dependent form of regulated cell death characterized by iron dyshomeostasis, glutathione depletion, glutathione peroxidase 4 dysfunction, and excessive lipid peroxidation. Exercise is a safe and accessible non-pharmacological intervention with broad neuroprotective potential, but the evidentiary basis linking exercise specifically to ferroptosis is uneven. Only a limited subset of studies directly combines an exercise intervention with ferroptosis-related outcomes in neurodegenerative models; much of the proposed pathway architecture is inferred from pharmacological, cellular, observational, or acute neurological injury studies. This review therefore separates direct exercise evidence from exercise-related supporting evidence and non-exercise mechanistic evidence. The most directly relevant findings, concentrated largely in aerobic exercise models, show exercise-associated changes in brain iron handling, the cystine/glutamate antiporter–glutathione peroxidase 4 antioxidant system, and lipoxygenase-dependent lipid peroxidation. Supporting studies suggest additional peripheral-to-central mechanisms involving muscle-derived exosomes, exercise-associated changes in systemic and cerebral iron handling, and inflammatory regulation. Bone marrow hematopoiesis, adult neurogenesis, synaptic plasticity, and astrocyte-controlled iron traffic are incorporated as biologically plausible but incompletely tested links. Evidence for resistance training, high-intensity interval training, mind–body exercise, and human disease remains insufficient. The central limitation is therefore not pathway plausibility but the scarcity of exercise-specific causal experiments demonstrating that ferroptosis suppression is required for neuroprotection.

## 1. Introduction

Neurodegenerative diseases are progressive and largely incurable disorders that erode cognition, movement, independence, and quality of life. Their clinical importance is magnified by population aging because several major disorders, particularly Alzheimer’s disease and Parkinson’s disease, become increasingly common in later life, while amyotrophic lateral sclerosis, Huntington’s disease, and multiple sclerosis contribute distinct patterns of motor, cognitive, and inflammatory disability [1]. Despite their heterogeneous phenotypes and initiating events, these diseases repeatedly converge on oxidative stress, mitochondrial dysfunction, neuroinflammation, and impaired protein homeostasis [2]. Because available therapies remain predominantly symptomatic and rarely halt neuronal loss, identifying shared and potentially modifiable mechanisms is a clinical and biological priority.

Among these convergent mechanisms, ferroptosis is of particular interest because it connects iron dyshomeostasis with failure to detoxify phospholipid hydroperoxides. detoxification. First described by Dixon et al. in 2012 [3], ferroptosis is characterized by glutathione depletion, glutathione peroxidase 4 (GPX4) dysfunction, and iron-dependent phospholipid peroxidation [4]. Increasing evidence implicates ferroptosis-related processes across neurodegenerative disorders [5,6]. This framework can link disease-specific pathology to a shared form of membrane injury: cerebral iron accumulation and weakened antioxidant defense accompany amyloid and tau pathology in Alzheimer’s disease [7], whereas substantia nigra iron may interact with α-synuclein aggregation in Parkinson’s disease [5,8]. Disturbed iron handling and ferroptosis-related lipid injury have also been implicated in amyotrophic lateral sclerosis and Huntington’s disease [9,10], while inflammatory demyelination and abnormal iron distribution provide a plausible, although less directly validated, context in multiple sclerosis [6]. Ferroptosis may therefore represent a convergent vulnerability, but its causal weight is unlikely to be identical across diseases, cell types, or disease stages.

Exercise is relevant to this problem not only as a broadly neuroprotective behavior but also as a systemic intervention capable of modifying several upstream determinants of ferroptotic vulnerability, including mitochondrial function, inflammatory tone, neurotrophic signaling, and peripheral-to-brain communication [11,12]. In preclinical models, exercise has altered transferrin receptor 1 (TfR1), ferroportin (FPN), and hepcidin [13,14,15,16] and has been associated with changes in solute carrier family 7 member 11 (SLC7A11), GPX4, and lipoxygenase-dependent lipid oxidation in Alzheimer’s and Parkinson’s disease models [15,17]. These findings create a credible mechanistic bridge between exercise adaptation and ferroptosis. The central controversy, however, is whether they demonstrate ferroptosis-dependent neuroprotection or merely parallel changes in broadly exercise-responsive antioxidant and inflammatory pathways. This uncertainty is amplified by the predominance of aerobic animal studies, the frequent reliance on isolated markers, and the limited evidence for other exercise modalities, human disease, and pathway-specific rescue.

A focused synthesis is therefore needed not simply to catalog exercise-responsive pathways, but to determine where the evidence chain is complete and where it remains inferential. This review evaluates exercise–ferroptosis interactions across major neurodegenerative diseases, exercise modalities, and mechanistic pathways. It distinguishes direct exercise-intervention evidence from exercise-related supporting evidence and non-exercise mechanistic findings, thereby clarifying which mechanisms are supported, which are plausible but unconfirmed, and which should be treated as testable hypotheses.

## 2. Methods

### 2.1. Literature Search

This narrative and mechanistic review was supported by a structured literature search and was not designed as a systematic review or meta-analysis. Relevant studies were identified through searches of PubMed, Web of Science Core Collection, and Scopus from database inception to 31 May 2026. The search focused on ferroptosis-related processes, major neurodegenerative diseases, exercise interventions, and exercise-responsive molecular mediators. Cognitive impairment terms were included to identify mechanistically relevant studies, but non-neurodegenerative cognitive-injury models were treated only as supporting or indirect evidence. Search terms included ferroptosis, iron metabolism, lipid peroxidation, antioxidant defense, GPX4, nuclear factor erythroid 2-related factor 2 (NRF2), SLC7A11, Alzheimer’s disease, Parkinson’s disease, amyotrophic lateral sclerosis, Huntington’s disease, multiple sclerosis, cognitive impairment, physical activity, aerobic exercise, resistance training, high-intensity interval training, exerkines, irisin, brain-derived neurotrophic factor (BDNF), and microRNAs. Terms within each conceptual domain were combined using “OR”, and the ferroptosis, disease, and exercise domains were combined using “AND”, with database-specific adaptations where required. Reference lists of relevant reviews and included studies were also screened manually.

### 2.2. Study Relevance and Selection

Studies were considered relevant if they: (i) examined ferroptosis-related mechanisms in neurodegenerative diseases or established experimental models of these diseases; (ii) evaluated the effects of exercise or physical activity on ferroptosis-related outcomes or pathways; or (iii) investigated exercise-responsive mediators with a biologically plausible link to ferroptosis regulation. Exercise-intervention studies constituted the primary evidence base. Studies of healthy animals, physiological aging, or acute neurological injury were retained only as exercise-related supporting evidence, whereas non-exercise pharmacological, cellular, genetic, and observational studies were included only to define mechanistic plausibility or evidence gaps. Publications unrelated to ferroptosis, neurodegeneration, exercise, or exercise-responsive molecular mediators, as well as conference abstracts, editorials, letters, book chapters, duplicate records, and non-peer-reviewed publications, were excluded. Studies reporting only general oxidative stress, inflammation, or neuroprotection without a defined connection to iron metabolism, lipid peroxidation, or ferroptosis-related pathways were not used as ferroptosis evidence. Review articles were used to contextualize the field and identify potentially relevant primary studies; mechanistic and intervention claims were based preferentially on original research.

### 2.3. Evidence Synthesis and Classification

**Figure 1:**
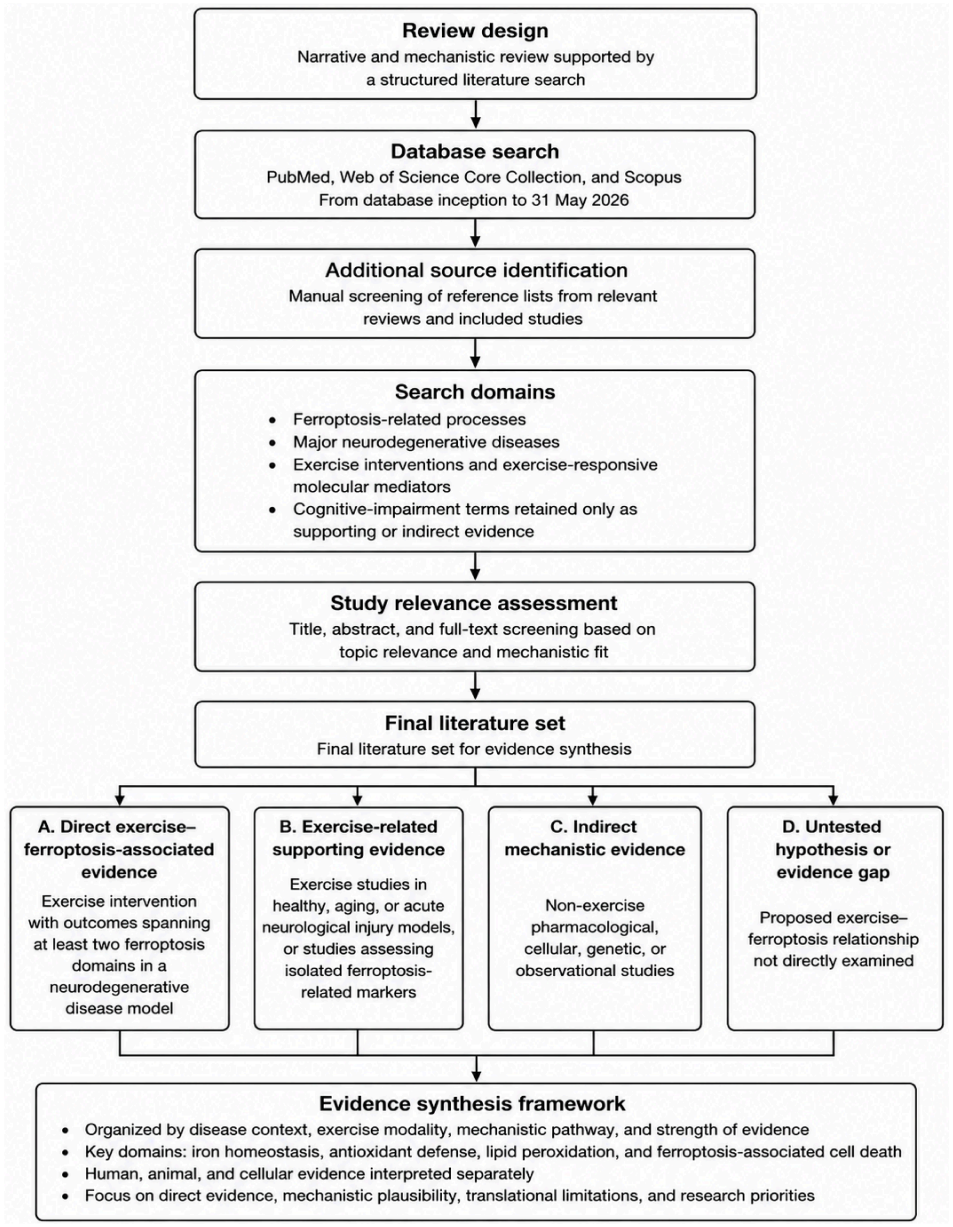
Structured literature search, study relevance assessment, and four-category evidence-classification framework.

The selected literature was synthesized narratively according to disease type, exercise modality, ferroptosis-related pathway, and strength of evidence. Evidence was assigned to one of four categories: (i) direct exercise–ferroptosis-associated evidence in neurodegenerative disease models, in which an exercise intervention was accompanied by outcomes spanning at least two ferroptosis domains in a neurodegenerative disease model; (ii) exercise-related supporting evidence, in which exercise altered isolated iron-handling, antioxidant-defense, lipid-peroxidation, or cell-death markers without sufficiently establishing ferroptotic cell death, or in which ferroptosis-related outcomes were examined in healthy, aging, or acute neurological injury models; (iii) indirect mechanistic evidence, derived from non-exercise pharmacological, cellular, genetic, or observational studies that identified biologically relevant pathways but did not test an exercise intervention; and (iv) an untested hypothesis or evidence gap, when the proposed exercise–ferroptosis relationship had not been experimentally examined. Evidence was considered causally stronger when studies assessed multiple ferroptosis domains, including iron dysregulation, glutathione/GPX4 antioxidant defense, lipid peroxidation, and ferroptosis-associated cell death, and when pathway dependence was demonstrated using genetic manipulation or pharmacological rescue with established ferroptosis inhibitors. Changes in a single marker, such as tissue iron content, malondialdehyde concentration, SLC7A11 expression, or GPX4 expression or activity, were not considered sufficient to establish ferroptotic cell death. Likewise, activation of a broadly cytoprotective pathway, such as NRF2 signaling, was not interpreted as evidence of ferroptosis regulation unless accompanied by ferroptosis-relevant downstream outcomes. This classification was applied throughout the text and evidence tables and informed the evidence-graded conceptual framework. The structured literature search, study-relevance assessment, and evidence-classification framework are summarized in Figure 1.

## 3. Disease-Specific Ferroptosis Mechanisms and Evidence Boundaries

### 3.1. Core Molecular Cascade and Evidentiary Criteria

Ferroptosis emerges when iron-dependent phospholipid oxidation exceeds the capacity of cellular defense systems [3,4]. The process begins upstream with iron handling: TfR1 and divalent metal transporter 1 facilitate iron uptake, whereas FPN supports iron export. Expansion of the intracellular ferrous-iron pool promotes Fenton chemistry and reactive oxygen species generation, thereby increasing the oxidative pressure on membrane phospholipids [6,18]. Whether this pressure becomes lethal depends on two opposing systems. On the protective side, SLC7A11 sustains cystine uptake and glutathione synthesis, GPX4 reduces phospholipid hydroperoxides, and NRF2 coordinates antioxidant and iron-storage genes, including SLC7A11, GPX4, and ferritin heavy chain 1 [19,20]. On the pro-ferroptotic side, acyl-CoA synthetase long-chain family member 4 (ACSL4) enriches membranes with polyunsaturated phospholipids that can undergo lipoxygenase-dependent oxidation [21]. The resulting imbalance produces the characteristic biochemical phenotype and may be accompanied by mitochondrial shrinkage, increased membrane density, and reduced cristae [6].

These molecular features also define the evidentiary threshold for attributing neuronal injury to ferroptosis. Brain iron dyshomeostasis establishes a plausible disease context [22], but stronger inference requires convergence across iron dysregulation, antioxidant failure, phospholipid peroxidation, compatible cellular or ultrastructural injury, and, ideally, rescue by an established ferroptosis inhibitor or genetic manipulation. Ferrostatin-1-sensitive death in 1-methyl-4-phenylpyridinium-exposed neuronal cells illustrates the added value of rescue-based evidence [23]. By contrast, pharmacological modulation of TrkB-related signaling and environmental enrichment show that ferroptosis-associated pathways are experimentally tractable [24,25], but they do not demonstrate that exercise recruits those pathways. Accordingly, an isolated change in tissue iron, malondialdehyde, SLC7A11, GPX4, or NRF2 should be described as ferroptosis-associated rather than as proof of ferroptotic neuronal death.

### 3.2. Alzheimer’s Disease: Iron–Proteinopathy Feedback and Antioxidant Failure

In Alzheimer’s disease, disrupted iron homeostasis is increasingly regarded as an active contributor to neuronal vulnerability rather than a passive by-product of degeneration. Iron accumulation has been observed in the vicinity of amyloid-β plaques, where excess iron may catalyze reactive oxygen species generation via the Fenton reaction and thereby promote membrane phospholipid peroxidation; in parallel, it may facilitate amyloid-β aggregation and abnormal tau phosphorylation [22]. These processes appear to reinforce one another: protein aggregation promotes local iron retention, retained iron intensifies oxidative injury, and this injury in turn aggravates amyloid and tau pathology. Apolipoprotein E genotype may further influence cerebral iron handling and ferritin turnover, whereas ferroptosis or ferroptosis-associated dysfunction in microglia may propagate injury to adjacent neurons through non-cell-autonomous mechanisms [26].

Impairment of the SLC7A11–glutathione–GPX4 defense system may further compound this vulnerability. In Alzheimer’s disease tissues and experimental models, reduced SLC7A11 expression may limit cystine uptake and deplete glutathione, thereby impairing GPX4-dependent reduction of phospholipid hydroperoxides and promoting the accumulation of lipid-peroxidation end-products such as 4-hydroxynonenal and malondialdehyde [7,21,27]. Iron accumulation, diminished detoxification of lipid peroxides, and oxidative membrane injury therefore converge to create a molecular environment consistent with increased ferroptotic vulnerability. It should be noted, however, that these markers also participate in broader oxidative-stress responses and can change in non-disease-specific cellular injury models [27]. In the absence of convergent biochemical, morphological, cellular, and rescue-based evidence, any individual change should be interpreted as a ferroptosis-associated finding rather than proof of ferroptotic neuronal death.

### 3.3. Parkinson’s Disease: Iron–α-Synuclein Feedback and Lipid-Defense Failure

In Parkinson’s disease, ferroptotic vulnerability can be understood as a convergence of regional iron loading, weakened detoxification of phospholipid hydroperoxides, and disturbed membrane remodeling. Iron accumulation in the substantia nigra increases pro-oxidant pressure, while reduced glutathione availability and impaired GPX4-dependent defense limit the removal of oxidized phospholipids [28]. Loss of phospholipase A2 group VI function can further disrupt membrane lipid remodeling and promote dopaminergic neuronal ferroptosis through the peroxiredoxin 6 (PRDX6)/ferritin heavy chain 1 (FTH1)/GPX4 axis [29]. Thus, iron provides the oxidative drive while failure of lipid repair lowers the threshold for neuronal injury.

This vulnerability may be amplified by a feed-forward interaction between iron and α-synuclein. Iron promotes α-synuclein aggregation and oxidative modification; aggregated α-synuclein, in turn, disrupts mitochondrial, vesicular, and iron-handling processes, thereby sustaining lipid peroxidation [8]. Evidence that 1-methyl-4-phenylpyridinium-induced neuronal death is sensitive to ferrostatin-1 strengthens the inference beyond marker coexistence [23]. α-Lipoic acid has likewise improved motor outcomes while attenuating ferroptosis-associated injury in a Parkinson’s disease model [30], although this remains pharmacological rather than exercise-derived evidence.

Taken together, the sequence from regional iron accumulation to impaired lipid-peroxide defense, α-synuclein amplification, and rescue-sensitive neuronal injury supports ferroptosis as a contributor to dopaminergic vulnerability, but not as the exclusive mechanism of neuronal death. Exercise-specific evidence involving SLC7A11, lipoxygenases, lipid oxidation, and microglial injury is therefore evaluated separately in Section 4, where it can be graded according to the number of ferroptosis domains measured and the presence or absence of pathway-specific rescue.

### 3.4. Amyotrophic Lateral Sclerosis: Motor-Neuron Vulnerability and Iron Dysregulation

In amyotrophic lateral sclerosis, the ferroptosis argument is centered on selective motor-neuron vulnerability. Ferroptosis inhibition preserves motor-neuron viability in experimental models, providing rescue-based evidence that is stronger than an isolated change in iron or lipid-peroxidation markers [9]. This vulnerability is biologically coherent with disturbed iron uptake, storage, and export: expansion of the labile iron pool can intensify oxidative and mitochondrial stress, particularly in superoxide dismutase 1 (SOD1)-associated disease models.

This sequence positions iron dyshomeostasis as a plausible upstream driver of ferroptotic susceptibility in amyotrophic lateral sclerosis, but important links remain unresolved. It is not yet clear how the TfR1/FPN axis connects to GPX4 failure, ferritinophagy, and oxidized-phospholipid accumulation across motor neurons and glial cells, or whether correcting iron handling is sufficient to prevent ferroptotic death. The exercise-responsive component of this pathway is considered in Section 4 rather than within the disease-pathology subsection.

### 3.5. Huntington’s Disease and Multiple Sclerosis: Plausible Mechanisms and Major Evidence Gaps

Mutant huntingtin can impair mitochondrial function, energy metabolism, iron homeostasis, and antioxidant defense, thereby increasing striatal neuronal susceptibility to iron-dependent lipid injury in Huntington’s disease [10]. Nevertheless, the existing evidence is derived mainly from disease-mechanism and therapeutic studies, and direct exercise interventions examining ferroptosis-related outcomes in Huntington’s disease have not been identified. Huntington’s disease is therefore included primarily as disease-related mechanistic context rather than as a condition with established exercise–ferroptosis evidence.

In multiple sclerosis, inflammatory demyelination, abnormal iron distribution, microglial activation, and oxidative injury to lipid-rich myelin membranes provide a biologically plausible context for ferroptosis-related damage [6]. Oligodendrocytes may be particularly susceptible because of their iron requirements and their role in producing lipid-rich myelin. However, the current reference list does not contain a primary study directly demonstrating exercise-mediated ferroptosis regulation in multiple sclerosis. The existing exercise literature primarily addresses disease progression and functional outcomes rather than ferroptosis-specific mechanisms [31]. Multiple sclerosis should therefore be presented as a mechanistically plausible but experimentally unvalidated exercise–ferroptosis context. The major ferroptosis-related molecular alterations and their pathological significance across neurodegenerative diseases are summarized in Table 1.

## 4. Exercise Evidence by Modality and Supporting Neurological Context

### 4.1. Aerobic Exercise: The Deepest Disease-Specific Evidence Chain

Within the current literature, aerobic exercise provides the only disease-specific evidence chain spanning more than one ferroptosis domain, consistent with the predominance of aerobic protocols in current mechanistic syntheses [32]. In amyloid precursor protein/presenilin-1 (APP/PS1) mice, eight weeks of moderate treadmill exercise improved cognition while reducing prefrontal cortical iron accumulation and shifting the system Xc^−^/GPX4 defense pathway toward greater lipid-peroxide control [15]. This finding links an upstream change in iron handling to restoration of antioxidant defense and a functional outcome, making it more informative than a single-marker study.

The Parkinson’s disease evidence extends this chain from bulk tissue to a defined cell type and lipid-peroxidation pathway. In 1-methyl-4-phenyl-1,2,3,6-tetrahydropyridine-treated mice, voluntary wheel running increased microglial SLC7A11, reduced arachidonate 12-lipoxygenase (ALOX12)-dependent lipid oxidation, attenuated microglial ferroptosis-associated injury, and improved dopaminergic and motor outcomes [17]. The Alzheimer’s and Parkinson’s disease studies therefore converge on complementary points of the ferroptosis network—iron/GPX4 defense and cell-specific lipid oxidation—although neither established that pathway blockade abolishes the neuroprotective effect of exercise. Swimming training in SOD1 mice provides a weaker but relevant extension: reduced spinal TfR1, increased FPN, and improved motor outcomes indicate exercise-responsive iron regulation, but ferroptotic death and pathway necessity were not demonstrated [16].

Aerobic exercise is therefore the best-supported modality because it has generated the deepest evidence chain, not because biological superiority over other modalities has been demonstrated. No head-to-head study has compared aerobic, resistance, high-intensity interval, combined, and mind–body exercise under matched workloads with ferroptosis-specific outcomes. Such comparisons should control for energy expenditure, duration, sex, age, and disease stage and should test pathway dependence rather than relying on isolated changes in iron, malondialdehyde, or GPX4.

### 4.2. Resistance Exercise: Mechanistic Plausibility Without Direct Validation

The evidence gap for resistance exercise is direct and substantial: no peer-reviewed study identified through the present search combined a resistance-training intervention with ferroptosis-related outcomes in a neurodegenerative disease model. Resistance exercise produces mechanical, anabolic, and muscle-secretory responses that differ from continuous aerobic exercise [32], creating a plausible route to altered brain iron handling or antioxidant defense, but plausibility is currently the beginning rather than the end of the evidence chain.

The closest supporting evidence comes from outside both neural tissue and exercise intervention. Irisin-enriched exosomes inhibited ferroptosis through caveolin-1 in osteoblasts [33], showing that a muscle-associated secreted factor can regulate ferroptosis under specific conditions. Because the study did not test resistance exercise or neuronal injury, it cannot establish a resistance-exercise–ferroptosis pathway in the brain.

Resistance training should therefore remain a hypothesis-generating modality until direct studies connect a defined training dose to central or spinal iron handling, antioxidant defense, phospholipid peroxidation, cell-type-specific injury, and ferroptosis-directed rescue. Such studies should report load, intensity, volume, and recovery and include matched sedentary controls.

### 4.3. High-Intensity Interval and Combined Exercise: Relevant Adaptations Without Direct Ferroptosis Evidence

Evidence for high-intensity interval training currently stops upstream of ferroptosis. In healthy mice, six weeks of high-intensity interval training increased lactate and hippocampal BDNF and modified mitochondrial quality-control proteins [34]. These responses may improve neuronal metabolism and plasticity, but the study did not assess brain iron handling, oxidized phospholipids, ferroptotic cell death, or pathway dependence.

Combined aerobic–resistance training is even less directly characterized. Its metabolic and mechanically induced muscle signals could theoretically converge on NRF2- and BDNF-related neuroprotection, and such a framework has been proposed for Parkinson’s disease [35]. However, the supporting publication is a mechanistic synthesis rather than a combined-training experiment, so it establishes a testable hypothesis rather than an intervention effect.

High-intensity interval and combined exercise should therefore not be described as validated ferroptosis-regulating modalities. Direct comparisons with continuous aerobic and resistance exercise are needed to determine whether their intensity profiles and endocrine responses produce distinct effects on central iron metabolism and ferroptosis-related injury.

### 4.4. Mind–Body Exercise: No Direct Ferroptosis Validation

For Tai Chi, yoga, and related mind–body exercise, the evidence chain is shorter still. These interventions have been associated with inflammatory and stress-related signaling [36], and NRF2/BDNF-mediated ferroptosis regulation has been proposed as a framework for exercise in Parkinson’s disease [35]. No identified study, however, combined a mind–body exercise intervention with ferroptosis-related outcomes in a neurodegenerative disease model. Their anti-ferroptotic role should therefore be presented as untested rather than inferred from general anti-inflammatory or neurotrophic effects.

### 4.5. Physiological and Aging Models: Adaptation Rather than Disease Evidence

Physiological and aging models answer a narrower question than disease models: they show whether exercise can remodel iron and redox biology before or outside established neurodegeneration. In wild-type animals, treadmill or voluntary running altered ferritin transcription, GPX4 expression, hepcidin, interleukin-6, and brain–muscle iron distribution [13,15]. In aged rats, aerobic training was associated with lower hippocampal iron, less tau-related pathology, and reduced oligodendrocyte ferroptosis-associated injury [14]. These convergent changes establish an exercise-responsive physiological substrate for ferroptosis regulation.

They do not, however, establish an anti-ferroptotic treatment effect in a defined chronic disease. Without disease-specific neuronal loss, convergent cell-death measures, and rescue-based validation, these findings should be classified as physiological or aging-related support rather than evidence that exercise modifies Alzheimer’s disease, Parkinson’s disease, or amyotrophic lateral sclerosis through ferroptosis.

### 4.6. Adjacent Non-Exercise Interventions and Acute Neurological Injury: Supporting Context

Adjacent non-exercise interventions demonstrate pathway tractability but not exercise specificity. Environmental enrichment attenuated ferroptosis-associated injury in chronic cerebral hypoperfusion [25], and repetitive transcranial magnetic stimulation altered ferroptosis-related outcomes in aging-associated cognitive impairment [37]. Because neither intervention is a defined exercise modality, these findings support the modifiability of ferroptosis-related pathways but cannot substantiate an exercise mechanism.

Acute neurological injury provides the converse form of support: the intervention is exercise, but the disease context is not chronic neurodegeneration. Treadmill training attenuated ferroptosis-related injury after cerebral ischemia–reperfusion through SLC7A11/GPX4 signaling [37], while moderate-intensity treadmill exercise reduced traumatic brain injury-associated ferroptosis through suppression of stimulator of interferon genes signaling [38]. These studies connect exercise to ferroptosis-related pathways in the injured brain, yet their acute time course, susceptible cell populations, and inflammatory environment limit transfer to slowly progressive disease. They should therefore remain supporting mechanistic evidence rather than direct evidence for chronic neurodegeneration. The available exercise-intervention and exercise-associated evidence across neurological models is summarized in Table 2.

## 5. Mechanistic Evidence Chains and Their Boundaries

### 5.1. Iron Homeostasis: The Best-Supported Upstream Link

Iron handling currently provides the best-supported upstream link between exercise and ferroptosis-related neuroprotection. Increased TfR1, reduced FPN-mediated export, and expansion of the labile Fe^2+^ pool can intensify Fenton chemistry and membrane lipid peroxidation in neurodegenerative conditions [39]. Exercise studies intervene at this entry point: treadmill training in APP/PS1 mice reduced cortical iron accumulation and TfR1 while increasing FPN [15], and swimming training produced the same TfR1-to-FPN shift in the spinal cord of superoxide dismutase 1 mice [16]. The convergence across Alzheimer’s disease and amyotrophic lateral sclerosis models supports exercise-responsive iron redistribution, although the latter study did not directly establish ferroptotic cell death.

Physiological and aging models extend this pattern but also define its boundary. Aerobic training reduced hippocampal iron accumulation together with oligodendrocyte ferroptosis-associated injury in aged rats [14], whereas voluntary running coordinated brain–muscle iron redistribution with lower cortical hepcidin and interleukin-6 in wild-type and 5xFAD mice [13]. These findings connect local iron transport to systemic inflammatory and iron-regulatory signals. However, because neither hepcidin nor the TfR1/FPN axis was selectively blocked, the studies do not show that this route is required for exercise-induced suppression of ferroptosis.

The evidence chain therefore progresses from an upstream mechanism, through reproducible exercise-responsive transport proteins, to parallel changes in ferroptosis-associated injury and function. It remains incomplete at the causal step: regional and cell-specific iron responses, dose dependence, and the necessity of iron redistribution for neuroprotection have not been adequately tested.

### 5.2. NRF2/SLC7A11/GPX4 Defense: Responsive but Not Yet Causally Required

Where iron handling determines oxidative pressure, the NRF2/SLC7A11/GPX4 system determines whether phospholipid hydroperoxides are removed before membrane injury becomes lethal. SLC7A11 sustains cystine uptake and glutathione synthesis, GPX4 uses glutathione to detoxify phospholipid hydroperoxides, and NRF2 coordinates this antioxidant response with iron-regulatory gene expression [19,20]. The key question is therefore not whether the system changes after exercise, but whether its activation is necessary for the observed neuroprotection.

The strongest disease-specific support comes from APP/PS1 mice, in which eight weeks of aerobic exercise increased system Xc^−^/GPX4 defense while reducing iron- and lipid-peroxidation-related injury [15]. A cerebral ischemia–reperfusion study produced a parallel response—greater SLC7A11/GPX4 signaling accompanied by less ferroptosis-related injury after treadmill training [40]—but represents acute-injury rather than chronic neurodegenerative evidence. Together, the studies show pathway responsiveness across two neurological contexts; neither used genetic disruption or ferroptosis-directed rescue to establish pathway necessity.

Non-exercise interventions reinforce the tractability of this defense system without closing the exercise-specific causal gap. Forsythoside A, senegenin, Schisandra lignans, and salidroside reduced ferroptosis-associated injury through NRF2- or GPX4-related signaling in Alzheimer’s disease-related, cellular, or cognitive-impairment models [7,27]. These findings establish biological relevance, but not recruitment by exercise. NRF2 activation or an isolated increase in GPX4 should therefore remain supporting evidence unless it converges with phospholipid-peroxidation, cell-death, and rescue-based outcomes.

### 5.3. Lipid Remodeling and Peroxidation: Limited Disease-Specific Evidence

Even when iron pressure increases and antioxidant defense weakens, ferroptosis still requires an oxidizable phospholipid substrate. ACSL4 expands this substrate pool, whereas lipoxygenases convert polyunsaturated phospholipids into ferroptosis-associated oxidation products [17,21]. This places lipid remodeling at the execution side of the proposed exercise–ferroptosis pathway, but exercise-specific evidence at this level is less developed than evidence for iron handling or GPX4 defense.

The clearest disease-specific chain was demonstrated in MPTP-treated mice. Voluntary wheel running increased microglial SLC7A11, reduced ALOX12 expression and arachidonic-acid oxidation products, attenuated microglial ferroptosis-associated injury, and improved dopaminergic and motor outcomes [17]. By linking a defined cell type to both antioxidant defense and lipid oxidation, this study advances beyond bulk-tissue marker reporting. Comparable evidence has not been established for resistance, high-intensity interval, combined, or mind–body exercise.

Danggui Shaoyao San and melatonin provide complementary non-exercise evidence that ACSL4-related lipid pathways can be modified in Alzheimer’s disease or cognitive-injury models [21,41]. Their effects support pathway tractability, but they cannot be used as evidence that exercise acts through ACSL4. The critical unresolved step is whether exercise changes specific oxidized phospholipid species and whether blocking ACSL4 or lipoxygenase-dependent oxidation alters the neurological benefit.

### 5.4. Peripheral-to-Brain Signaling: A Causal Example and Unconfirmed Candidates

Peripheral-to-brain signaling offers a mechanism by which a systemic exercise stimulus could alter neuronal ferroptotic vulnerability. The most complete causal example comes from cerebral ischemia: exercise preconditioning increased skeletal muscle-derived exosomal microRNA-484, which suppressed neuronal ACSL4, reduced lipid peroxidation, and increased SLC7A11 and GPX4; inhibiting muscle microRNA-484 weakened these protective effects [42]. This sequence connects a defined peripheral source to a central target, downstream ferroptosis domains, and loss-of-function evidence.

The remaining candidate mediators occupy earlier positions in the evidence chain. Irisin reduced ferroptosis-related injury in high-glucose-exposed hippocampal cells [43], and irisin-enriched exosomes inhibited osteoblast ferroptosis through caveolin-1 [33], but neither experiment traced an exercise signal from muscle to the diseased brain. Likewise, high-intensity interval training increased lactate and hippocampal BDNF [34], while voluntary running altered interleukin-6, hepcidin, and brain–muscle iron distribution [13]. TrkB modulators can also exert receptor-independent anti-ferroptotic activity [24], illustrating why a change in BDNF/TrkB signaling alone cannot identify a ferroptosis-specific route. These findings nominate myokines, metabolites, cytokines, iron hormones, and extracellular vesicles; they do not yet establish an exercise-dependent anti-ferroptotic pathway in chronic neurodegeneration.

The microRNA-484/ACSL4 study therefore supplies the strongest causal peripheral-to-brain example, but its acute cerebral-ischemia setting limits disease transferability. Whether the same muscle–exosome–neuron sequence operates in Alzheimer’s disease, Parkinson’s disease, or amyotrophic lateral sclerosis remains untested.

### 5.5. Bone Marrow Hematopoiesis: A Potential Myeloid Bridge

Bone marrow hematopoiesis could form a systemic bridge between exercise and the brain by modifying the number and phenotype of inflammatory cells to which neural tissue is exposed. In mice, voluntary running reduced hematopoietic stem and progenitor cell activity and chronic leukocyte production while preserving emergency hematopoiesis; reduced adipose leptin signaling contributed to progenitor quiescence [44]. Recent evidence further suggests that exercise can attenuate myeloid skewing during maintenance hematopoiesis under metabolic stress [45]. These findings indicate that exercise can modulate hematopoietic activity and myeloid output under selected experimental conditions.

Ferroptosis may influence this process by shaping hematopoietic stem-cell survival and lineage bias. Human hematopoietic stem cells are susceptible to ferroptosis [46], whereas ferroptosis resistance has been associated with the maintenance and myeloid differentiation of long-term hematopoietic stem cells through pathways involving SLC7A11/GPX4 signaling and ferritinophagy [47]. These observations raise the possibility that ferroptosis-resistant, myeloid-biased stem-cell populations could sustain inflammatory myeloid output under metabolic or inflammatory stress.

A separate line of human genetic and neuropathological evidence links clonal hematopoiesis to Alzheimer’s disease biology and identifies blood-derived mutations in brain myeloid-cell-enriched fractions [48]. Together, these findings support a testable sequence in which exercise limits hematopoietic activation and myeloid skewing, potentially reducing the peripheral inflammatory pressure experienced by the brain. However, no study has yet shown that exercise alters myeloid output by modifying ferroptosis sensitivity in hematopoietic stem cells. Bone marrow hematopoiesis should therefore remain a hypothesis-generating bridge rather than direct exercise–ferroptosis evidence. Future studies should combine exercise with lineage-resolved hematopoietic profiling, ferroptosis measurements in defined stem-cell subsets, myeloid-cell tracking, and pathway-specific rescue.

### 5.6. Adult Neurogenesis and Synaptic Plasticity: Downstream Hypotheses

Adult neurogenesis and synaptic plasticity are plausible downstream consequences of reduced ferroptotic stress, but they are not yet established components of an exercise–ferroptosis pathway. In neural stem cells from 5xFAD mice, increased lipid reactive oxygen species and reduced GPX4 coincided with impaired neuronal differentiation and lower synaptosomal-associated protein 25; GPX4 overexpression improved lipid-peroxide defense and neuronal-lineage differentiation [49]. This experiment links ferroptosis control to neurogenic competence, but contains no exercise intervention.

In vivo evidence strengthens the same downstream relationship: hippocampal neural precursor cells showed age-dependent ferroptosis susceptibility, and modifying that vulnerability altered neurogenesis and memory [50]. The cellular and in vivo studies therefore converge on a ferroptosis-to-neurogenesis link. Because neither tested exercise, they cannot establish the reverse inference that exercise-induced neurogenesis is mediated by ferroptosis suppression.

The missing experiment is a dependency test: exercise should be combined with lineage tracing, electrophysiology, oxidized-phospholipid profiling, and genetic or pharmacological manipulation of ferroptosis to determine whether ferroptosis suppression is required for neurogenesis, long-term potentiation, or synaptic remodeling.

### 5.7. Astrocyte-Controlled Iron Traffic: A Cell-Specific Hypothesis

Astrocytes provide a cell-specific route through which systemic exercise signals could influence both cerebral iron entry and neuronal antioxidant capacity. Astrocyte-derived hepcidin regulates FPN on brain microvascular endothelial cells, and astrocyte-specific hepcidin deficiency caused brain iron overload and cognitive impairment in mice [51]. Because astrocytes also support neuronal cysteine and glutathione availability, they could couple blood–brain barrier iron traffic to GPX4-dependent lipid-peroxide detoxification.

A Parkinson’s disease model adds a propagation step to this hypothesis: disruption of oligodendrocyte–astrocyte fibroblast growth factor signaling was followed first by preferential astrocytic ferroptosis and then by dopaminergic neuronal ferroptosis [52]. Astrocytes may therefore act both as regulators of iron traffic and as targets or transmitters of ferroptotic stress.

**Figure 2:**
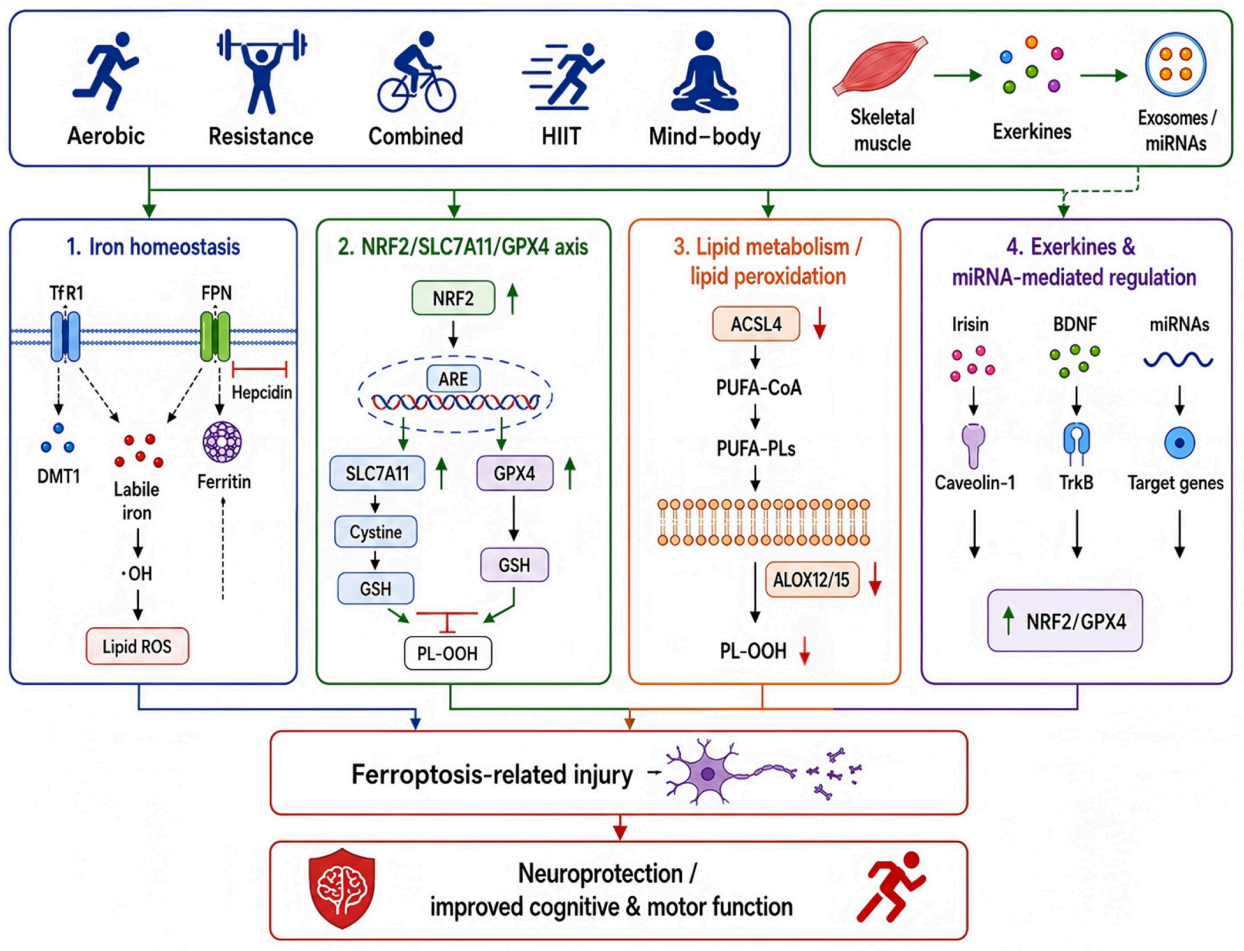
Proposed mechanistic framework linking exercise to ferroptosis-related neuroprotection. Exercise may influence ferroptotic vulnerability through four interrelated domains: iron homeostasis, the NRF2/SLC7A11/GPX4 antioxidant axis, lipid metabolism and lipid peroxidation, and peripheral-to-brain signaling mediated by exerkines and microRNAs. Together, these pathways may reduce ferroptosis-related injury and contribute to neuroprotection and improved cognitive and motor function. Solid arrows indicate established or proposed pathway relationships, whereas dashed arrows indicate indirect or incompletely validated interactions. Green upward arrows indicate exercise-associated increases in protective factors, whereas red downward arrows indicate exercise-associated decreases in pro-ferroptotic factors and lipid-peroxidation products. Abbreviations: ACSL4, acyl-CoA synthetase long-chain family member 4; ALOX12/15, arachidonate lipoxygenase 12/15; ARE, antioxidant response element; BDNF, brain-derived neurotrophic factor; DMT1, divalent metal transporter 1; FPN, ferroportin; GSH, glutathione; GPX4, glutathione peroxidase 4; HIIT, high-intensity interval training; miRNAs, microRNAs; NRF2, nuclear factor erythroid 2-related factor 2; PL-OOH, phospholipid hydroperoxide; PUFA-CoA, polyunsaturated fatty acyl-coenzyme A; PUFA-PLs, polyunsaturated fatty acid-containing phospholipids; SLC7A11, solute carrier family 7 member 11; system Xc^−^, cystine/glutamate antiporter system; TfR1, transferrin receptor 1; TrkB, tropomyosin receptor kinase B.

The exercise-specific link remains missing. No direct study has shown that astrocytic hepcidin, antioxidant support, lipid handling, or ferroptotic susceptibility mediates exercise-induced neuroprotection. Cell-type-resolved experiments are therefore required to distinguish astrocytic responses from those of neurons, microglia, oligodendrocytes, and endothelial cells. The relative strength of evidence across these proposed mechanisms is summarized in Table 3, and their integrated mechanistic relationships are illustrated in Figure 2.

## 6. Translational Limitations and Research Priorities

### 6.1. Evidence Depth and Ferroptosis Validation

The first translational limitation is evidence depth. Only a small number of studies directly combine exercise, ferroptosis-related outcomes, and a chronic neurodegenerative disease model, and most use treadmill or voluntary running [13,14,15,17,33,34,35,53]. Resistance, high-intensity interval, combined, and mind–body exercise therefore appear less supported primarily because they have been studied less often. The present literature can rank evidence volume, but it cannot establish the biological superiority of aerobic exercise.

The second limitation is validation depth. Changes in iron, malondialdehyde, reactive oxygen species, NRF2, SLC7A11, or GPX4 are compatible with ferroptotic vulnerability but are not individually diagnostic of ferroptotic cell death [3,4,19,20]. A stronger design should connect iron dysregulation to phospholipid peroxidation, antioxidant failure, cell viability, and compatible morphology, and should then test reversibility through genetic manipulation or an established ferroptosis-directed rescue. This convergence is essential because every isolated marker also participates in broader oxidative-stress or inflammatory responses.

### 6.2. Model Transferability and Human Translation

Model transferability forms a separate problem. Healthy animals, physiological aging, cerebral ischemia, and traumatic brain injury show that exercise can alter iron and ferroptosis-related pathways [13,14,38,40], but their disease duration, susceptible cell populations, inflammatory environment, and timing of tissue injury differ from chronic neurodegeneration. These models can establish biological plausibility; they cannot substitute for disease-modifying evidence in Alzheimer’s disease, Parkinson’s disease, amyotrophic lateral sclerosis, or other progressive disorders.

The same inference problem becomes more pronounced in humans, where direct assessment of neuronal ferroptosis in the living brain is difficult. Circulating iron indices, oxidative-stress markers, and inflammatory mediators cannot by themselves represent central ferroptotic death. Human studies should therefore triangulate iron-sensitive neuroimaging, cerebrospinal-fluid or blood biomarkers, oxidized-lipid profiling, and disease-specific functional outcomes. Peripheral measurements will be most informative when interpreted alongside central nervous system measures rather than as independent surrogates of brain ferroptosis.

### 6.3. Exercise-Dose Standardization

Even a validated ferroptosis outcome cannot resolve modality differences if the exercise dose is poorly specified. Existing protocols vary in intensity, duration, frequency, progression, and total workload, making it difficult to separate the effect of exercise type from the effect of training dose. Comparative studies should match workload or energy expenditure and report adherence, physiological intensity, and recovery. Sex, age, disease stage, baseline activity, and metabolic comorbidity should be incorporated because each can modify iron metabolism, antioxidant capacity, inflammation, and exercise adaptation.

### 6.4. Mechanistic Priorities for Future Studies

The central research priority is to convert plausible links into tested dependencies. Hepcidin, inflammatory cytokines, lactate, irisin, and extracellular vesicles can connect peripheral exercise responses to the brain, but causal evidence in chronic neurodegeneration remains limited [13,33,34,42,43,44]. Bone marrow hematopoiesis, neurogenesis, synaptic plasticity, and astrocyte-controlled iron traffic are still more distal hypotheses. A specific priority is to determine whether exercise reduces pro-inflammatory myeloid output by altering ferroptosis sensitivity in myeloid-biased hematopoietic stem-cell subsets. Future studies should therefore intervene at a defined node, resolve the responding cell type, and determine whether disrupting ferroptosis regulation diminishes the neurological benefit of exercise. This design would replace parallel marker changes with a causal evidence chain.

## 7. Conclusions

Exercise consistently intersects with three determinants of ferroptotic vulnerability—iron homeostasis, defense against phospholipid hydroperoxides, and lipid oxidation—but the strength of the evidence decreases along the causal chain. Selected aerobic disease models connect exercise to multiple ferroptosis domains and neurological outcomes; most other modalities and supporting models provide only partial or indirect links. The current literature therefore does not establish ferroptosis suppression as a consistent, generalizable, or necessary mechanism of exercise-induced neuroprotection.

Ferroptosis should consequently be viewed as a plausible and testable mechanistic bridge, not yet as a confirmed exercise target. Progress will depend on designs that connect a defined exercise dose to systemic signals, cell-specific ferroptosis regulation, rescue-sensitive neuronal outcomes, and clinically relevant function. Until those links are reproduced in human disease, ferroptosis-related measures should guide mechanistic investigation rather than exercise prescription.

## Figures and Tables

**Table 1:** Major ferroptosis-related molecular alterations and their pathological significance in neurodegenerative diseases.

Molecule/ Pathway	Typical Alteration	Mechanistic Role	Disease Context	Biological Significance	Evidence Context
TfR1	↑ Increased or upregulated	Promotes transferrin-bound iron uptake and increases intracellular iron availability	AD, PD, and ALS models	May expand the labile iron pool and increase susceptibility to Fenton chemistry and lipid peroxidation	Animal models and disease-related mechanistic studies
FPN	↓ Reduced or dysregulated	Limits cellular iron export and promotes intracellular iron retention	AD, PD, and ALS models	Impaired iron efflux may aggravate neuronal or glial iron accumulation and oxidative stress	Animal models and iron-homeostasis studies
Ferritin/ferritinophagy	↓ Reduced storage capacity or dysregulated ferritin turnover	Alters iron sequestration and may increase the release of redox-active iron	AD, PD, and ALS-related models	May enlarge the labile iron pool and promote oxidative injury	Human tissue, animal, and cellular studies
Labile Fe^2+^	↑ Increased	Participates in Fenton reactions and promotes hydroxyl-radical formation	AD, PD, ALS, and HD-related models	Provides an upstream source of iron-dependent oxidative stress and membrane lipid peroxidation	General ferroptosis mechanisms and disease models
system Xc^−^/SLC7A11	↓ Reduced or suppressed	Decreases cystine uptake and limits glutathione synthesis	AD and PD-related models	Weakens antioxidant capacity and increases vulnerability to lipid-peroxide accumulation	Animal and cellular models
GSH	↓ Depleted	Serves as an essential cofactor for GPX4-mediated reduction of phospholipid hydroperoxides	AD, PD, and related experimental models	Compromises lipid-peroxide detoxification and lowers the threshold for ferroptotic injury	Human tissue, animal, and cellular studies
GPX4	↓ Reduced expression or activity	Reduces phospholipid hydroperoxides to less reactive phospholipid alcohols	AD, PD, ALS, and related neurological models	Loss of GPX4-dependent defense permits accumulation of oxidized membrane phospholipids	Human tissue and experimental models
NRF2	↓ Impaired activation or nuclear translocation	Coordinates antioxidant, iron-storage, and detoxification genes, including SLC7A11, GPX4, and FTH1	AD and PD-related models	Reduced activity may broadly weaken antioxidant and anti-ferroptotic defenses	Mainly pharmacological, cellular, and animal evidence
ACSL4	↑ Increased expression or activity	Promotes incorporation of polyunsaturated fatty acids into membrane phospholipids	AD, PD, and related experimental models	Increases oxidizable phospholipid substrates and ferroptotic sensitivity	Mainly animal, cellular, and non-exercise studies
ALOX12/15	↑ Increased expression or activity	Promotes oxidation of polyunsaturated phospholipids	AD, PD, and experimental neurological models	Facilitates accumulation of oxidized phospholipids associated with ferroptotic injury	Animal and cellular mechanistic studies
ROS	↑ Increased	Promotes mitochondrial dysfunction, oxidative damage, and membrane lipid oxidation	PD and multiple related models	Amplifies interactions among iron dysregulation, oxidative stress, and lipid peroxidation	Broad oxidative-stress and ferroptosis-related evidence
FSP1/CoQ10 pathway	↓ Reduced activity or insufficient protection	Regenerates reduced CoQ10 and suppresses lipid peroxidation independently of GPX4	AD-related and experimental models	Weakens GPX4-independent protection against membrane lipid oxidation	Mainly cellular and mechanistic evidence; disease-specific evidence remains limited

Abbreviations: ACSL4, acyl-CoA synthetase long-chain family member 4; AD, Alzheimer’s disease; ALOX12/15, arachidonate lipoxygenase 12/15; ALS, amyotrophic lateral sclerosis; CoQ10, coenzyme Q10; Fe^2+^, ferrous iron; FPN, ferroportin; FSP1, ferroptosis suppressor protein 1; FTH1, ferritin heavy chain 1; GPX4, glutathione peroxidase 4; GSH, glutathione; HD, Huntington’s disease; NRF2, nuclear factor erythroid 2-related factor 2; PD, Parkinson’s disease; ROS, reactive oxygen species; SLC7A11, solute carrier family 7 member 11; system Xc^−^, cystine/glutamate antiporter system; TfR1, transferrin receptor 1. ↑ indicates an increase or upregulation, whereas ↓ indicates a decrease or downregulation. Note: The listed alterations summarize patterns reported across human tissue, animal, and cellular studies. A change in a single marker is not sufficient to establish ferroptotic cell death without convergent biochemical, morphological, cellular, or rescue-based evidence.

**Table 2:** Evidence map of exercise interventions and exercise-associated studies relevant to ferroptosis in neurological models.

Exercise Intervention	Model	Protocol	Ferroptosis-Related Findings	Main Interpretation	Disease Relevance	Evidence Status
Treadmill aerobic exercise	APP/PS1 mice	8 weeks, moderate intensity	Reduced cortical Fe^2+^ and TfR1; increased FPN and GPX4	Modified iron transport and the system Xc^−^/GPX4 defense pathway	Alzheimer’s disease	Direct exercise–ferroptosis-associated evidence; causal pathway dependence not established
Voluntary wheel running	MPTP-treated mice	6 weeks	Reduced ALOX12 and lipid oxidation; increased SLC7A11; attenuated microglial ferroptosis-associated changes	Cell-specific association between exercise, lipid-peroxidation regulation, and microglial vulnerability	Parkinson’s disease	Direct exercise–ferroptosis-associated evidence in a neurodegenerative model
Swimming training	SOD1 mice	8 weeks	Reduced spinal TfR1; increased FPN and AKT signaling	Modified spinal iron handling and motor outcomes	Amyotrophic lateral sclerosis	Exercise-related supporting evidence; ferroptotic cell death and pathway dependence not established
Voluntary wheel running	Wild-type and 5xFAD mice	Long-term running	Altered brain–muscle iron distribution; reduced cortical hepcidin and cortical/circulating interleukin-6	Coordinated peripheral and central iron adaptation	Healthy and Alzheimer’s disease models	Exercise-related supporting evidence; no direct ferroptosis endpoint
Treadmill aerobic exercise	Aged rats	8 weeks	Reduced hippocampal iron and tau-related pathology; attenuated oligodendrocyte ferroptosis-associated changes	Aging-associated adaptation of iron handling and oligodendrocyte injury	Physiological aging	Exercise-related supporting evidence from an aging model
Treadmill exercise	Mice with traumatic brain injury	4 weeks, moderate intensity	Increased GPX4; reduced lipid-derived reactive oxygen species; suppressed STING signaling	Attenuated ferroptosis-related injury after traumatic brain injury	Acute neurological injury	Exercise-related supporting evidence from an acute neurological injury model
Preconditioning exercise	Rats with cerebral ischemia	Exercise before ischemic injury	Muscle-derived exosomal miR-484 suppressed ACSL4, reduced lipid peroxidation, and increased SLC7A11/GPX4	Causally informative muscle-to-brain exosomal pathway	Acute neurological injury	Causally informative exercise–ferroptosis evidence from an acute neurological injury model
High-intensity interval training	Healthy mice	6 weeks	Increased lactate and hippocampal BDNF; modified mitochondrial quality-control proteins; ferroptosis not measured	Metabolic and neuroplastic adaptation without direct ferroptosis assessment	Healthy model	Exercise-related mechanistic evidence without a ferroptosis endpoint

Evidence status: direct exercise–ferroptosis-associated evidence required an exercise intervention and outcomes spanning multiple ferroptosis-related domains in a neurodegenerative model. Supporting evidence included studies with isolated markers or studies conducted in healthy, aging, or acute neurological injury models. A single-marker change was not considered sufficient to establish ferroptotic cell death. Abbreviations: ACSL4, acyl-CoA synthetase long-chain family member 4; AKT, protein kinase B; ALOX12, arachidonate 12-lipoxygenase; APP/PS1, amyloid precursor protein/presenilin-1; BDNF, brain-derived neurotrophic factor; Fe^2+^, ferrous iron; FPN, ferroportin; GPX4, glutathione peroxidase 4; MPTP, 1-methyl-4-phenyl-1,2,3,6-tetrahydropyridine; miR-484, microRNA-484; SLC7A11, solute carrier family 7 member 11; SOD1, superoxide dismutase 1; STING, stimulator of interferon genes; system Xc^−^, cystine/glutamate antiporter system; TfR1, transferrin receptor 1.

**Table 3:** Evidence hierarchy for the proposed mechanisms linking exercise to ferroptosis-related neuroprotection.

Mechanistic Dimension	Current Exercise Evidence	Main Evidence Gap	Evidence Level
Iron homeostasis	Exercise modifies TfR1, FPN, hepcidin, and brain-peripheral iron distribution.	The causal requirement of iron redistribution for ferroptosis suppression remains untested.	Direct and supporting evidence
Antioxidant defense	Exercise alters the NRF2/SLC7A11/GPX4 system in neurodegenerative and acute-injury models.	Genetic or pharmacological confirmation of pathway dependence is limited.	Direct and supporting evidence
Lipid remodeling and peroxidation	Exercise reduces lipoxygenase-related lipid oxidation in a Parkinson’s disease model.	Evidence is concentrated in one disease model, with limited data on ACSL4 and oxidized phospholipids.	Limited direct evidence
Peripheral-to-brain signaling	Exercise-derived exosomal and metabolic signals can regulate ferroptosis-related pathways in acute neurological injury.	Translation to chronic neurodegenerative diseases has not been demonstrated.	Supporting evidence
Emerging systemic and cellular mechanisms, including myeloid output	Exercise reduces hematopoietic activity and may attenuate myeloid skewing; neurogenesis and astrocyte-mediated iron regulation remain plausible links.	Whether exercise alters myeloid output through ferroptosis regulation in hematopoietic stem cells remains untested.	Indirect evidence and testable hypotheses

Note: Evidence levels follow the definitions provided in Section 2. A change in a single ferroptosis-related marker was not considered sufficient to establish ferroptotic cell death. Abbreviations: ACSL4, acyl-CoA synthetase long-chain family member 4; FPN, ferroportin; GPX4, glutathione peroxidase 4; NRF2, nuclear factor erythroid 2-related factor 2; SLC7A11, solute carrier family 7 member 11; TfR1, transferrin receptor 1.

## Data Availability

No new data were created or analyzed in this study. Data sharing is not applicable to this article.

## Abbreviations

The following abbreviations are used in this manuscript:

ACSL4	acyl-CoA synthetase long-chain family member 4
ALOX12	arachidonate 12-lipoxygenase
APP/PS1	amyloid precursor protein/presenilin-1
BDNF	brain-derived neurotrophic factor
FPN	ferroportin
GPX4	glutathione peroxidase 4
GSH	glutathione
MPTP	1-methyl-4-phenyl-1,2,3,6-tetrahydropyridine
NRF2	nuclear factor erythroid 2-related factor 2
SLC7A11	solute carrier family 7 member 11
SOD1	superoxide dismutase 1
system Xc^−^	cystine/glutamate antiporter system
TfR1	transferrin receptor 1
